# Phytochemical properties and health benefits of pregelatinized Tartary buckwheat flour under different extrusion conditions

**DOI:** 10.3389/fnut.2022.1052730

**Published:** 2022-11-09

**Authors:** Zhuo Zhang, Xin Fan, Liang Zou, Bao Xing, Manli Zhu, Xiushi Yang, Guixing Ren, Yang Yao, Lizhen Zhang, Peiyou Qin

**Affiliations:** ^1^Key Laboratory of Chemical Biology and Molecular Engineering of Ministry of Education, School of Life Sciences, Shanxi University, Taiyuan, China; ^2^Key Laboratory of Quality Evaluation and Nutrition Health of Agro-Products, Ministry of Agriculture and Rural Affairs, Institute of Crop Sciences, Chinese Academy of Agricultural Sciences, Beijing, China; ^3^Key Laboratory of Coarse Cereal Processing, Ministry of Agriculture and Rural Affairs, Sichuan Engineering & Technology Research Center of Coarse Cereal Industrialization, School of Food and Biological Engineering, Chengdu University, Chengdu, China; ^4^Institute of Bast Fiber Crops, Chinese Academy of Agricultural Sciences, Changsha, China

**Keywords:** Tartary buckwheat, extrusion, flavonoids, antioxidant activity, α-glucosidase inhibitory activity, α-amylase inhibitory activity

## Abstract

This work investigated the phytochemical properties and health benefits of Tartary buckwheat flour obtained with different extrusion conditions including high, medium, and low temperature. Extrusion significantly decreased the fat content and changed the original color of Tartary buckwheat flour. The contents of protein, total flavonoids, and D-*chiro*-inositol were affected by the extrusion temperature and moisture. Extrusion significantly decreased the total flavonoids and flavonoid glycosides contents, while it significantly increased aglycones. Compared to native Tartary buckwheat flour and pregelatinization Tartary buckwheat flour obtained with traditional extrusion processing technology, the pregelatinization Tartary buckwheat flour obtained with improved extrusion processing technology contained higher aglycones and lower flavonoid glycosides, which had stronger antioxidant capacity, α-glucosidase inhibitory activity and relatively mild α-amylase inhibitory activity. Correlation analysis proved that the aglycone content was positively correlated with antioxidant and α-glucosidase inhibitory activities. These findings indicate that the pregelatinization Tartary buckwheat flour obtained with improved extrusion processing technology could be used as an ideal functional food resource with antioxidant and anti-diabetic potential.

## Introduction

Tartary buckwheat [*Fagopyrum tataricum* (L.) Gaench], which is a species of buckwheat, has been cultivated since ancient times, and is currently distributed in Asia (especially in China), America, and Europe (1). As a pseudocereal, Tartary buckwheat has been receiving increasing attention as a potential functional ingredient or food that is rich in a range of nutrients including bioactive carbohydrates and proteins, polyphenols, phytosterols, flavonoids, D-*chiro*-inositol (DCI), vitamins, carotenoids, and minerals. These nutritious substances endow Tartary buckwheat with various health benefits such as antioxidant, anti-cancer, anti-diabetic, anti-hypertensive, cholesterol-lowering, and anti-inflammatory properties (2–4). Our previous study reported that flavonoids are the main phenolic secondary metabolites in Tartary buckwheat grain, and they are 10-fold higher than in common buckwheat (5). The flavonoids mainly include rutin, quercetin, and kaempferol and their glycoside forms, of which rutin is the most abundant in Tartary buckwheat (6–8). Moreover, Tartary buckwheat is an important natural source of DCI, which is a compound with an insulin-like bioactivity (9). Previous studies have indicated that DCI has a synergistic effect with phenolic compounds in the treatment of type II diabetes. Specifically, DCI mainly eliminates insulin resistance by enhancing the body’s sensitivity to insulin, thereby regulating blood glucose (10, 11), and the phenolic compounds mainly control blood glucose by inhibiting the activity of α-glucosidase and α-amylase (12). However, strong α-amylase inhibitory activity can cause abnormal fermentation by bacteria in the colon, triggering a series of adverse reactions (such as diarrhea and flatulence). Therefore, the recommended way to control blood sugar is to have strong α-glucosidase inhibitory activity and relatively mild α-amylase inhibitory activity (13, 14).

Based on above nutritional and functional properties, many products related to Tartary buckwheat have been developed, such as noodles, tea, baked goods, and meal replacement powder (1). However, native Tartary buckwheat flour (NTBF) is gluten-free and cannot form an optimal network structure, which restricts the development of Tartary buckwheat products and their sensory quality (13, 15). Therefore, in order to increase the utility of Tartary buckwheat in the food processing industry, several processing technologies have been used to modify NTBF including high pressure (15), high hydrostatic pressure (16), fermentation (8, 17) and roasting, boiling, extrusion, and microwave treatment (3, 18). These treatment methods have been shown to change the phytochemical composition of Tartary buckwheat, thereby affecting its nutritional properties. Our previous research found that the rutin in Tartary buckwheat degraded into quercetin and rutinoside during processing (5). Although the high concentration of quercetin has stronger antioxidant and α-glucosidase inhibitory properties than rutin, it will produce a bitter taste and decrease the sensory quality (4, 8, 19). Therefore, antioxidant and α-glucosidase inhibition as well as the sensory qualities and levels of bitterness should always be taken into account when processing Tartary buckwheat foods. It has been confirmed that hydrothermal treatment of Tartary buckwheat at 100°C for 20 min can better maintain the rutin and quercetin content (17). To prevent rutin from degrading and thus reduce bitterness, Wu et al. found that superheated steam and saturated steam could efficiently inactivate rutin-degrading enzymes (RDEs) in Tartary buckwheat (20). High temperature wet heat treatment (cooking and extrusion) will inactivate most of the RDEs, while dry heat treatment has almost no effect on RDEs (21).

Among the processing methods considered above, extrusion cooking is a technology with high production efficiency, strong applicability, low cost, and energy consumption, and it has been widely used in the food industry (22, 23). Traditional extrusion processing technology (TEPT) is a continuous high-temperature short-term process that is frequently used to produce puffed food (23, 24). Nevertheless, previous studies have shown that TEPT significantly decreases antioxidant activity (AC) in buckwheat (25, 26). Recently, an improved extrusion processing technology (IEPT) was designed with a longer screw, lower speed, lower temperature, and higher pressure than TEPT (24). Buckwheat samples obtained with IEPT have shown a high retention rate of functional ingredients and desired physical properties (13).

At present, there is no systematic research on the changes to individual flavonoids, DCI, α-glucosidase, and α-amylase inhibitory activities in Tartary buckwheat, nor on the correlation between individual flavonoids and their biological functions after extrusion (TEPT and IEPT). Therefore, the aim of this study was to identify changes to phytochemical composition including nutritional substances, total and individual flavonoids, DCI, and color properties as well as the antioxidant, α-glucosidase, and α-amylase inhibitory activities in Tartary buckwheat samples after treatment with different extrusion conditions. A correlation analysis between individual flavonoids and their biological functions was also conducted in this study.

## Materials and methods

### Materials

Standard reagents (rutin, isoquercitrin, quercetin, and kaempferol), α-glucosidase (100 U), and p-nitrophenyl-a-D-glucopyranoside were purchased from the Yuan Ye Biological Technology Company (Shanghai, China); kaempferol-3-*O*-rutinoside and DNS reagent were purchased from Solarbio (Beijing, China); Trolox, a-amylase, 1,1-diphenyl-2-pic-rylhydrazyl (DPPH), 2,2’-azino-bis (3-ethylbenzothiazoline-6-sulphonic acid) (ABTS) and 2,4,6-tripyridyl-s-triazine (TPTZ) reagents were purchased from Sigma-Aldrich (Shanghai, China). Acarbose, used as a positive control, was produced by Bayer HealthCare Company Co. Ltd. (Beijing, China). The other chemicals and reagents used in this study were of analytical grade.

Dehulling of Tartary buckwheat groats were performed in Chengdu University. Native Tartary buckwheat flour (NTBF) was obtained by grinding using an experimental mill (Glen Creston Ltd., Stanmore, England) and passed through a 60-mesh sieve.

### Pre-gelatinization of Tartary buckwheat flour by different extrusion treatments

The Tartary buckwheat flour was extruded using a twin-screw extruder (Brabender KETSE 20/40, Duisburg, Germany) with a screw diameter of 20 mm, L/D ratio of 40:1 and die diameter of 1 mm. We used five of the six independent zones (we did not use the die) and controlled the temperature in the barrel in each. The samples were processed under different conditions as shown in Table 1. After extrusion, the samples were immediately oven-dried at 45°C for 24 h and then ground and sieved through 60 mesh to obtain pre-gelatinization of Tartary buckwheat flour (PTBF). All samples were stored at 4°C for further analysis. A total of 7 PTBF sample types were prepared and analyzed. They were denoted as TxMy, where x is the extrusion temperature at the fourth zone and y is the feed moisture content.

**TABLE 1:** Conditions of different extrusion treatments.

Treatments	Moisture content (%)	Extrusion temperate (°C)	The feeding rate (g/min)	The screw speed (rpm)
TEPT	30	40/75/110/160/95	40	100
IEPT	30, 40, 60	40/60/75/100/85	10	30
	30, 40, 60	40/60/70/70/70	10	30

TEPT, traditional extrusion processing technology; IEPT, improved extrusion processing technology.

### Chemical composition analysis

The compositions of Tartary buckwheat components including moisture, ash, protein, and fat were determined according to the methods of GB5009.3–2016, 5009.4–2016, 5009.5–2016, 5009.6–2016. The total starch content was determined by kit assays (Megazyme International Ireland Ltd., Wicklow, Ireland).

### Color determination

The color parameters of the Tartary buckwheat samples were measured following the method of Xiao et al. (8) with slight modifications. The colorimeter was calibrated using a standard white plate. Thirty replicate measurements were performed before the color parameters were recorded. The total color difference (°E), which represents the color change between PTBF and NTBF, was calculated using the following formula:


(1) $$△E\sqrt{{\left(L^{*}-L_{0}^{*}\right)}^{2}+{\left(a^{*}-a_{0}^{*}\right)}^{2}+{\left(b^{*}-b_{0}^{*}\right)}^{2}}$$

where L*_0_, a*_0_, and b*_0_ are the color parameters of NTBF; L*, a*, and b* are the color parameters of PTBF. L* means lightness (0 for black and 100 for white), a* is red (+) to green (−), and b* is yellow (+) to blue (−).

### Analysis of total flavonoids and individual flavonoid compounds

The total flavonoids and individual flavonoid compounds were extracted and determined according to a procedure described by our laboratory (5). The total flavonoids content (TFC) results were determined using the aluminum chloride colorimetric method and expressed as micrograms of rutin equivalent per gram of sample.

After the samples were passed through a 0.45 μm PEC syringe filter membrane (Jinteng, Tianjin, China), the individual flavonoid compounds including rutin, isoquercitrin, kaempferol-3-*O*-rutinoside, quercetin, and kaempferol were analyzed by an Alltech high performance liquid chromatography (HPLC) system (Alltech, Chicago, IL, USA) according to the method of Xiao et al. (8) with modifications. The analytical column was a PerkinElmer^®^ column (250 mm × 4.6 mm, Sheiton, USA) and the wavelength of the UV detector was set at 375 nm. The mobile phase was 0.05% trifluoroacetic acid aqueous solution (A) and 100% acetonitrile (B). A gradient flow system was established as follows with a flow rate of 1 ml/min: 0–8 min, 28% B; 8–18 min, 28–50% B; 18–30 min, 50–100% B; 30–35 min, 100% B; 35–38 min, 100–28% B; and 38–45 min, 28% B. The column temperature was kept at 30°C and the injection volume was 20 μl.

### Analysis of D-*chiro*-inositol

D-*chiro*-inositol in Tartary buckwheat samples was determined following the method established by our laboratory (27). Briefly, the sample (1 g) was mixed with 20 ml of 50% ethanol and incubated at room temperature for 30 min in a water bath with continuous shaking. The extract was passed through qualitative filter paper (Newstar, Hangzhou, China) and 1 ml of supernatant was transferred into a vial and oven-dried at 50°C. The dried extract was re-dissolved in 1 ml of methanol and passed through a 0.45 μm PEC syringe filter membrane (Jinteng, Tianjin, China) for immediate HPLC-ELSD (evaporative light scattering detector) analysis. A Prevail Carbohydrate ES 5u column (250 mm × 4.6 mm, Alltech, Chicago, IL, USA) was used. The injection volume was 10 μl. The mobile phase was 80% acetonitrile, the flow rate was set at 1 ml/min, and the eluant was sent to the ELSD (Alltech, Chicago, IL, USA). The temperature of the drift tube was set at 95°C, the nebulizing gas flow rate was 2.2 L/min, and the gain was 1.

### Antioxidant activity

The antioxidant activity of the flavonoids extracts from different Tartary buckwheat samples was evaluated by the following three methods with different reaction mechanisms.

#### 1,1-diphenyl-2-pic-rylhydrazyl radical scavenging capacity

1,1-diphenyl-2-pic-rylhydrazyl free radical scavenging capacity was determined following the method established by our laboratory (27). Sample solution (1 ml) was mixed with an equal volume of DPPH solution (0.4 mM) and incubated in darkness at room temperature for 20 min. The absorbance of the mixture was read at λ = 517 nm. Distilled water was used as a blank control. Trolox was measured at different concentrations (0–70 μg/ml) to produce a standard curve. The results were expressed as the Trolox equivalent concentration.

#### ABTS radical scavenging capacity

The ABTS activity was carried out as reported previously (27). Briefly, the ABTS stock solution was prepared by mixing equal volumes of ABTS preparation solution (7 mM) with potassium persulfate solution (2.45 mM) after incubation in the dark at room temperature for 16 h. The prepared solution was diluted with methanol until the absorbance was around 0.7 (±0.02) at λ = 734 nm. Next, the solution (20 μl) was mixed with 1,980 μl of ABTS working solution and incubated in darkness at room temperature for 6 min, and the absorbance was then determined at λ = 734 nm. Methanol was used as a blank control. Trolox was measured at different concentrations (10–100 μg/ml) to produce a standard curve. The results were expressed as the Trolox equivalent concentration.

#### Fe^3+^ reducing antioxidant power capacity

The Fe^3+^ reducing antioxidant power (FRAP) activity was performed according to the described method (8) with small modifications. The FRAP reagent was generated by mixing 300 mM sodium acetate buffer (pH 3.6), 10 mM TPTZ and 20 mM ferric chloride solution in 40 mM hydrochloric acid at a ratio of 10: 1: 1 (v/v/v). Sample solutions (200 μl) were mixed with 2 ml of FRAP reagent, and then incubated in a water bath at 37°C for 30 min. The absorbance was read at λ = 593 nm. Distilled water was used as a blank control. Trolox was measured at different concentrations (10–100 μg/ml) to produce a standard curve. The results were expressed as the Trolox equivalent concentration.

### α-glucosidase inhibition study *in vitro*

The α-glucosidase inhibitory activity of flavonoids extracts isolated from Tartary buckwheat samples was assessed by using the method of Xiao et al. (8). Before the experiment, the α-glucosidase (0.2 U/ml) was prepared by mixing it with phosphate buffer solution (PBS, 0.1 M, pH 6.8), and the sample solution (50 μl) was then mixed with 120 μl of the α-glucosidase solution. After incubation at 37°C for 10 min, 120 μl 2.5 mM 4-nitrophenyl-α-D-gluco-pyranoside (pNPG) in PBS was added. Then the mixture was incubated at 37°C for 15 min and the reaction was terminated by adding 480 μl 0.2 M sodium carbonate. Finally, the absorbance was measured at λ = 405 nm. The PBSZZ and acarbose were used as a reagent blank and positive control, respectively. The α-glucosidase inhibitory activity was calculated using the following formula:


(2) $$\text{Inhibition }\left(\%\right)=\left({\text{A}}_{0}-{\text{A}}_{\text{1}}\right)/{\text{A}}_{0}\times \text{1}00\;$$

where A_0_ and A_1_ represent the absorbance of the control and the experimental samples, respectively.

The α-glucosidase inhibitory activities of samples were represented as half inhibition concentration (IC_50_) values, meaning that 50% of α-glucosidase activity was inhibited at this concentration.

### α−amylase inhibition study *in vitro*

The α-amylase inhibitory activity of flavonoids extracts isolated from Tartary buckwheat samples was conducted according to the method described by Ji et al. (28). Specifically, sample solutions (100 μl) of different concentrations were mixed with 100 μl of α-amylase solution (4.5 U/ml) dissolved in PBS. After incubation at 37°C for 10 min, 100 μl of 1% (m/v) soluble starch (Xilong Chemical Factory Co., Ltd., Shantou, China) in PBS was added to start the reaction and further incubated at 37°C for 5 min. Next, 750 μl of DNS reagent was added to terminate the reaction, and the mixture immediately heated in a boiling water bath for 10 min. Afterward, the reaction mixture was immediately cooled down to room temperature and diluted three times. The absorbance was measured at λ = 540 nm, and sodium citrate buffer and acarbose were used as the reagent blank and positive control, respectively. The α-amylase inhibitory activity was calculated using equation (2). The α-amylase inhibitory abilities of the samples were shown as inhibiting percentages at 400 μg/ml, and the concentration of acarbose is 10 μg/ml.

### Statistical analysis

All analyses were carried out in triplicate; and the data are presented as mean ± standard deviation (SD). All the data were analyzed using SPSS 22.0. Analysis of variance (ANOVA) and Duncan’s multiple range tests (*p* < 0.05) were used to express the statistical significance of differences. The correlation matrix analysis was analyzed with the Pearson correlation coefficient.

## Results and discussion

### Nutritional composition

The nutritional composition of NTBF and PTBF are shown in Table 2. The total starch content of NTBF was 77.26 g/100 g dry weight (DW), and had higher values than in our previous studies (5, 29). This was due to an improved kit assay (AOAC Method 996.11 with a slight modification) used in this study that employed a thermostable α-amylase that is active and stable at pH 5. This modification is known to give higher starch values than those obtained with AOAC Method 996.11 (30). After extrusion, the total starch content of PTBF slightly increased or decreased, and ranged from 75.61 to 79.30 g/100 g DW. Similar results were observed in barley (31). Compared to NTBF, the protein content of PTBF obtained with TEPT and T100My significantly decreased (*p* < 0.05), while it increased in the PTBF obtained with T70My (*p* < 0.05). Previous studies have indicated that extrusion could cause the loss of protein and amino acids (32, 33). A previous kinetic study on the loss of lysine and other amino acids during extrusion of maize grits showed that the first-order rate constants were dependent mainly on extrusion temperature and feed moisture, whereas screw speed had no influence (34). Liu et al. (24) also found that a significant increase (*p* < 0.05) in protein was observed in texturized rice after adding 4% rice bran prior to treatment with IEPT. However, further studies are required to elucidate the mechanism of the increase in protein with IEPT. Compared to NTBF, the PTBF samples have a lower level of fat, and there was no significant difference (*p* > 0.05) in the ash content. These results are in agreement with an earlier study on brown rice (35). The decrease in fat content may be attributed to the interaction between lipid and amylose during extrusion (35).

**TABLE 2:** Concentrations of moisture, total starch, protein, fat, and ash contents of native and pregelatinized Tartary buckwheat flour (g/100 g dry weight).

Material	Moisture	Total starch	Protein	Fat	Ash
Native	10.98 ± 0.018b	77.26 ± 1.01b	11.47 ± 0.43cd	2.82 ± 0.08a	2.76 ± 0.19a
T160M30	5.92 ± 0.009g	79.09 ± 1.20a	10.95 ± 0.13f	0.85 ± 0.07d	2.76 ± 0.13a
T100M30	7.04 ± 0.033f	78.28 ± 0.33ab	11.11 ± 0.05ef	1.56 ± 0.12b	2.62 ± 0.28a
T100M40	7.40 ± 0.087e	79.30 ± 1.66a	11.43 ± 0.06de	1.49 ± 0.03b	2.66 ± 0.16a
T100M60	11.27 ± 0.119a	75.61 ± 0.25c	11.80 ± 0.13bc	1.07 ± 0.09c	2.66 ± 0.19a
T70M30	10.05 ± 0.058c	76.75 ± 0.51bc	11.98 ± 0.01b	0.52 ± 0.08e	2.62 ± 0.24a
T70M40	9.74 ± 0.047d	77.63 ± 0.39ab	12.07 ± 0.14b	1.00 ± 0.06c	2.63 ± 0.04a
T70M60	10.14 ± 0.059c	79.04 ± 0.67a	12.59 ± 0.27a	1.11 ± 0.11c	2.68 ± 0.27a

TxMy, x is the extrusion temperature at the fourth zone (°C) and y is the feed moisture content (%). The results were expressed as mean ± SD (*n* = 3) and different letters in the same column indicate significant differences (*p* < 0.05).

### Color attributes

The color parameters (L*, a*, b*, Δ*E*) are shown in Table 3. Different extrusion treatments had significant effects on the color parameters of Tartary buckwheat samples (*p* < 0.05), and the color manifestation is due to various mechanisms including non-enzymatic browning (such as Maillard reaction), pigment degradation and oxidation of ascorbic acid during the extrusion process (36, 37). NTBF was lighter than PTBF according to a decrease in the lightness (L*) score, which is consistent with a previous study (3). The free amino group of the amino acid can react with reducing sugars to form Maillard browning products during extrusion (3). Similarly, the a* scores of extruded samples significantly decreased (*p* < 0.05), except for T160M30 and T70M30. Conversely, the b* scores were significantly increased by the extrusion process, which means the color of PTBF was yellower than NTBF. Similarly observations have been reported by previous work, and they were explained by pigment degradation and non-enzymic browning reactions (38). ΔE is used to evaluate the overall color change, and generally, when Δ*E* values exceed 5.0, it is considered to be significantly different from the color of the control (37). The Δ*E* values of PTBF ranged from 10.34 to 34.27 (Table 3), indicating that extrusion affected the overall color of Tartary buckwheat samples. Therefore, the extrusion process significantly changes the original color of Tartary buckwheat.

**TABLE 3:** The color properties of the native and pregelatinized Tartary buckwheat flour.

Material	*L* *	*a* *	*b* *	Δ*E*
Native	79.92 ± 0.09a	0.18 ± 0.01c	20.06 ± 0.09f	–
T160M30	70.18 ± 0.10d	0.70 ± 0.01b	23.68 ± 0.59e	18.46 ± 0.50e
T100M30	75.15 ± 0.14c	−1.05 ± 0.04e	23.18 ± 0.34e	10.34 ± 0.34g
T100M40	75.44 ± 0.26c	−1.73 ± 0.02g	24.92 ± 0.03d	12.19 ± 0.33f
T100M60	66.39 ± 0.13f	−1.01 ± 0.02e	29.31 ± 0.38c	29.12 ± 0.49c
T70M30	64.57 ± 0.57g	2.26 ± 0.03a	29.56 ± 0.13c	32.22 ± 0.73b
T70M40	76.53 ± 0.12b	−0.88 ± 0.02d	30.59 ± 0.22b	19.69 ± 0.30d
T70M60	68.65 ± 0.09e	−1.23 ± 0.04f	35.71 ± 0.22a	34.27 ± 0.39a

TxMy, x is the extrusion temperature at the fourth zone (°C) and y is the feed moisture content (%). Data are expressed as means ± SD (*n* = 30) and different letters in the same column indicate significant differences (*p* < 0.05).

### Extractable total flavonoids and individual flavonoid compounds

The contents of total flavonoids and individual flavonoid compounds are presented in Table 4. All the extrusion processes significantly decreased (*p* < 0.05) TFC and flavonoid glycosides (rutin, isoquercitrin, kaempferol-3-*O*-rutinoside), while they significantly increased aglycones (quercetin and kaempferol). Sun et al. also reported that the extruded buckwheat flours showed lower contents of total flavonoids and rutin, as well as higher levels of quercetin than native flours (3). This study found that TFC was mainly affected by extrusion temperature and feed moisture. The loss of total flavonoids decreased with increases in the moisture content under the same extrusion temperature. When the moisture content was the same, the TFC in T70My was higher than T100My, but lower than T160M30. Similar results were also obtained by Cheng et al. (13). The TFC mainly depends on changes in the five individual flavonoid compounds, as shown in Table 4. On the one hand, most of rutin, isoquercitrin, kaempferol-3-*O*-rutinoside in PTBF obtained with IEPT was degraded to quercetin and kaempferol by flavonol-3-glucosidase (f3g) (8). On the other hand, it has been reported that high temperature processes (for instance, steaming) could cause the destruction of flavonoid compounds (8). This study also indicated that the sums of individual flavonoids in the different flours all had varying degrees of decline under different extrusion conditions.

**TABLE 4:** Concentrations of total flavonoids, DCI, and individual flavonoids in native and pregelatinized Tartary buckwheat flour (mg/g dry weight).

Material	Total flavonoids	DCI	Rutin	Isoquercitrin	Kaempferol-3-*O*-rutinoside	Quercetin	Kaempferol
Native	19.93 ± 0.35a	1.71 ± 0.08b	18.40 ± 0.23a	0.052 ± 0.014a	1.50 ± 0.12a	0.07 ± 0.00f	0.12 ± 0.01f
T160M30	17.61 ± 0.00b	1.79 ± 0.10ab	15.42 ± 0.53b	0.058 ± 0.002a	1.18 ± 0.06b	0.18 ± 0.01e	0.24 ± 0.01e
T100M30	11.81 ± 0.82f	1.90 ± 0.10a	0.64 ± 0.06de	0.016 ± 0.001c	0.05 ± 0.00d	4.70 ± 0.03d	0.54 ± 0.02c
T100M40	13.53 ± 0.56e	1.67 ± 0.12b	0.83 ± 0.03d	0.001 ± 0.000d	0.07 ± 0.00d	5.75 ± 0.05c	0.56 ± 0.00c
T100M60	14.91 ± 0.02d	1.14 ± 0.09d	0.86 ± 0.03d	ND	0.07 ± 0.00d	7.96 ± 0.05a	0.64 ± 0.01a
T70M30	13.89 ± 0.46e	1.49 ± 0.05c	3.82 ± 0.11c	0.026 ± 0.002b	0.25 ± 0.01c	4.79 ± 0.03d	0.50 ± 0.01d
T70M40	15.56 ± 0.12d	1.24 ± 0.05d	0.36 ± 0.03ef	ND	ND	6.94 ± 0.18b	0.61 ± 0.01b
T70M60	16.67 ± 0.24c	1.11 ± 0.04d	ND	ND	ND	8.21 ± 0.05a	0.61 ± 0.00b

TxMy, x is the extrusion temperature at the fourth zone (°C) and y is the feed moisture content (%). DCI, D-*chiro*-Inositol; ND, not detected. The results were expressed as mean ± SD (*n* = 3) and different letters in the same column indicate significant differences (*p* < 0.05).

**FIGURE 1:**
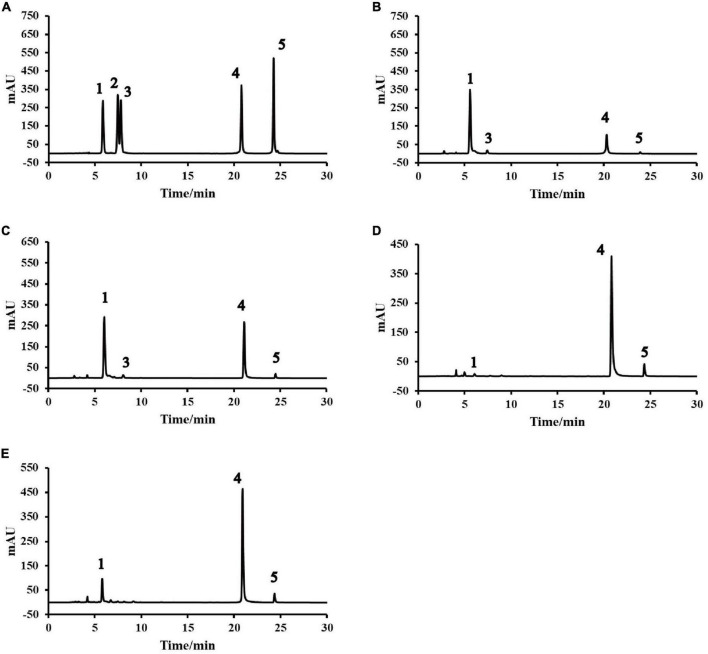
High performance liquid chromatography (HPLC) profiles of native and pregelatinized Tartary buckwheat flour: **(A)** Standards. **(B)** Native. **(C)** T160M30. **(D)** T100M30. **(E)** T70M30. (1) Rutin. (2) Isoquercitrin. (3) Kaempferol-3-*O*-rutinoside. (4) Quercetin. (5) Kaempferol. TxMy, x is the extrusion temperature at the fourth zone (°C) and y is the feed moisture content (%).

As shown in Table 4 and Figure 1, rutin had the highest retention rate at high temperature of 83.8% (15.42 mg/g DW). This result was in line with results obtained by Li et al. (21). This may be attributed to nearly complete denaturing of the rutin-degrading enzyme under high temperature conditions (8). For IEPT, when the temperature was kept constant and the feed moisture was increased from 30 to 60%, a significant (*p* < 0.05) increase was observed in flavonoid aglycones. The T70M60 sample had the highest proportion of aglycones (8.82 mg/g DW), while the glycosides are under the limit of detection. These results suggested that the moisture content of feed is an important factor for converting flavonoid glycosides to aglycones (especially quercetin). Additionally, moist heat is more destructive, and moisture produces a synergistic effect alongside high temperatures (22), which may be responsible for flavonoid glycoside degradation. Our previous study also reported that soak-treating Tartary buckwheat seeds significantly decreased the rutin content, whereas it significantly increased the contents of quercetin and kaempferol (5). Li et al. found that 96.46% of the rutin in the Tartary buckwheat dough was degraded into quercetin when the Tartary buckwheat flour was mixed with 50% water for 1 min at room temperature (21). In addition, Suzuki et al. (38) and Xiao et al. (8) has proved that f3g is an important enzyme involved in the transformation of the flavonoid compounds. Based on the above results, we hypothesized that different extrusion conditions including feed moisture content, temperature, feed rate, and screw speed could affect the activity of f3g and lead to changes in flavonoid content and composition. Further mechanistic studies are needed to elucidate this hypothesis.

### D-*chiro*-inositol content

Besides flavonoid compounds, changes in the DCI content of Tartary buckwheat samples obtained with different extrusion conditions was evaluated using a simple and rapid method based on HPLC linked to an evaporative light-scattering detector (HPLC-ELSD). As shown in Table 4, the DCI content in NTBF was 1.71 mg/g DW. For PTBF, the values of the DCI content ranged from 1.11 to 1.90 mg/g DW. Compared to NTBF, T100M30, and T160M30 samples had higher DCI levels (1.90 mg/g DW, *p* < 0.05; 1.79 mg/g DW, *p* > 0.05). Our previous study indicated that steaming buckwheat bran in an autoclave at 1.6 MPa and 120°C for 60 min could significantly enrich the DCI level in Tartary buckwheat bran extract from 0.03 to 0.22% (39). Similarly, Zielinski et al. (39) reported that baking at 220°C for 30 min significantly enhanced the DCI levels in buckwheat biscuits. Therefore, the likely explanation is that the high temperature and pressure caused by extrusion or other thermal processing technologies is able to disrupt galactosidic bonds and release the free form of DCI. Although other extrusion conditions (for instance T100M40, T100M60, and T70My) significantly decreased the DCI level (*p* < 0.05), the DCI level was still of the same order of magnitude (>1 mg/g DW). In addition, the mechanism of decrease was unclear.

### Antioxidant activity

The AC of bioactive components was related to a variety of determination methods with different mechanisms, including hydrogen atom transfer (HAT), single electron transfer (ET), reducing power, and metal chelation (40). Pellegrini et al. (41) reported that ABTS radicals are suitable for water and organic phases, whereas DPPH radicals can only be used for organic phases. In addition, FRAP assay was used to determine the AC of hydrophilic antioxidants with ferric reducing potency at acidic pH but it has a low sensitivity toward antioxidants with thiol-group. Therefore, the approach of using only one detection method may underestimate AC values because antioxidant compounds are incompletely extracted.

**FIGURE 2:**
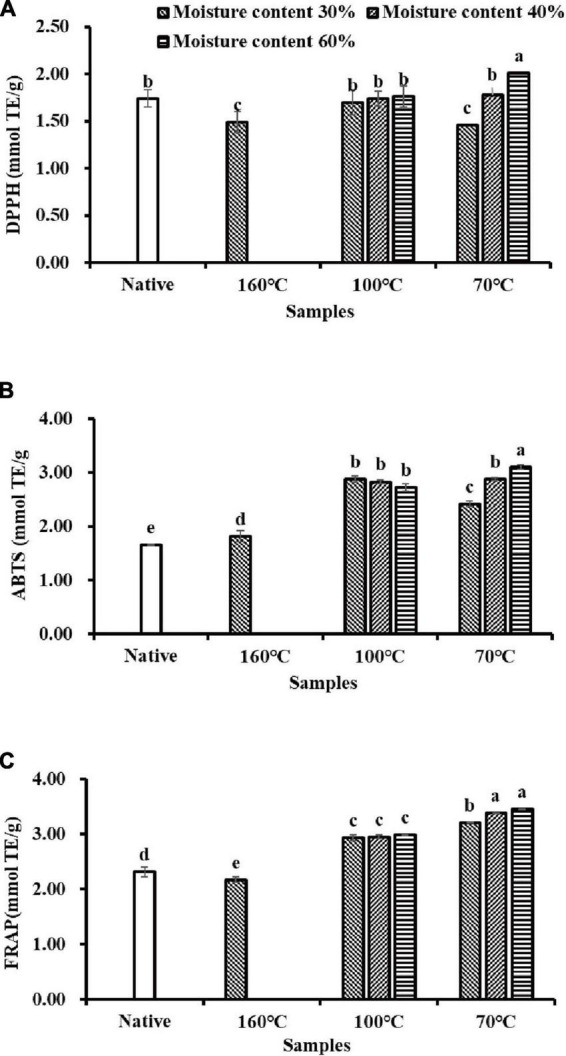
The antioxidant activities of flavonoids extracts isolated from native and pregelatinized Tartary buckwheat flour: **(A)** 1,1-diphenyl-2-pic-rylhydrazyl (DPPH) radical scavenging activity. **(B)** ABTS radical scavenging activity. **(C)** Ferric reducing antioxidant power (FRAP).

Three different assays including DPPH, ABTS, and FRAP were employed to evaluate the AC of flavonoid extracts isolated from Tartary buckwheat samples in this study. As shown in Figure 2A, DPPH radical scavenging activity in NTBF was 1.74 mmol Trolox equivalent (TE)/g DW, which was significantly higher (*p* < 0.05) than in the T160M30 and T70M30 samples, and significantly lower than T70M60 (*p* < 0.05). Moreover, DPPH radical scavenging activity of T100My and T70M40 samples showed no significant differences from NTBF (*p* > 0.05). The results shown in Figure 2B suggested that PTBF (especially samples produced by IEPT) possessed significantly (*p* < 0.05) higher ABTS^+^ radical-eliminating capacity than NTBF (1.66 mmol TE/g DW). In addition, the antioxidant capacity in PTBF determined with the ABTS assay was generally significantly higher than that determined with the DPPH assay, which is in agreement with previous studies (8, 40). This suggested the PTBF were rich in both lipophilic and hydrophilic radical scavengers. Similar results were observed in plant extracts (42). Compared to NTBF, the FRAP capacity of the T160M30 sample significantly decreased (*p* < 0.05), whereas PTBF obtained with IEPT showed a significant increase in the FRAP capacity (*p* < 0.05) (Figure 2C).

Combining the three detection methods above, these results confirmed that extrusion processes (especially samples produced by IEPT) significantly increased the AC (*p* < 0.05), Similar observations have also been reported in barley (22). The antioxidant compounds in the flavonoid extracts were not only good reductants but also efficient radical scavengers (42). The AC was contrary to the trend in changes in the flavonoid content. On the one hand, many studies have indicated that quercetin and kaempferol contain immensely higher AC than their glycoside compounds (8, 43). As mentioned before, flavonoid glycosides (rutin, isoquercitrin, kaempferol-3-*O*-rutinoside) were transformed into these aglycones (quercetin, kaempferol) after extrusion (especially in IEPT). Consequently, the AC of PTBF obtained with IEPT was much higher than in the non-extrusion treatment and TEPT treatment. Previous workers have also noted that quercetin-3-glucoside exhibits lower antioxidant capacity and it was proposed that this phenomenon was mainly due to the substitution of sugar or alkoxy groups that hinder the hydroxyl group (44). On the other hand, another study reported that thermal processing can produce pigments due to Maillard browning, and that these pigments enhance the antioxidant activity of the extrudate (22). To summarize, there are two possible explanations for improving the antioxidant activity: one was an alteration of the antioxidant profile and generation of more aglycones with stronger biological activities, and the other was due to contributions by the Maillard reaction.

### α-glucosidase and α-amylase inhibitory activities

**FIGURE 3:**
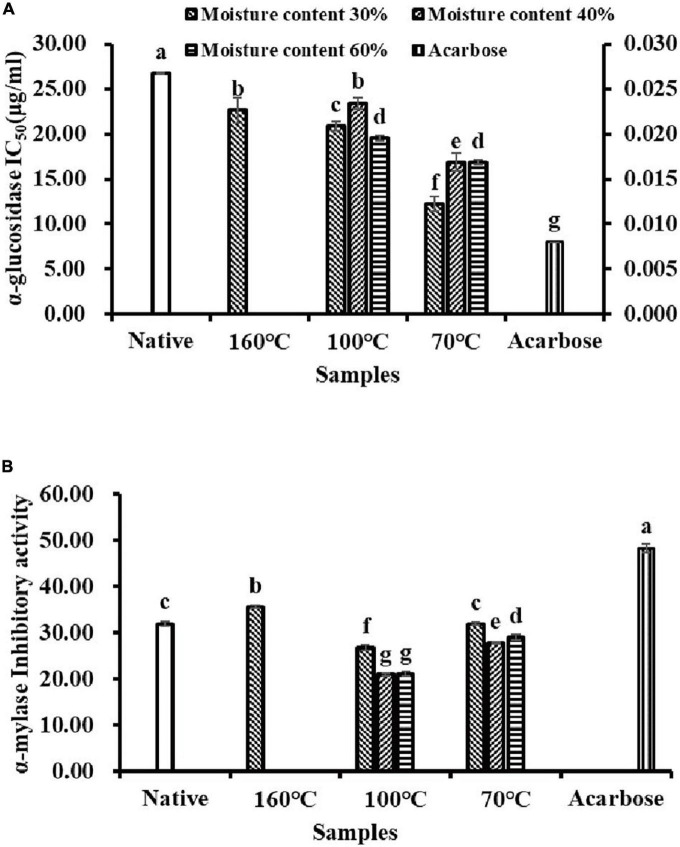
The α-glucosidase **(A)** and α-amylase **(B)** inhibitory activities of flavonoids extracts isolated from native and pregelatinized Tartary buckwheat flour.

The inhibitory effects of flavonoid extracts from Tartary buckwheat samples on α-glucosidase and α-amylase activities were investigated *in vitro* (Figure 3). Calculation of the half inhibitory concentration (IC_50_) of α-glucosidase was used to evaluate the inhibitory activity. As shown in Figure 3A, NTBF showed the lowest α-glucosidase inhibitory activity with the highest IC_50_ value (26.73 μg/ml). The α-glucosidase inhibitory activity of flavonoid extracts in PTBF was significantly enhanced by extrusion, with the IC_50_ values ranging from 12.23 to 23.45 μg/ml. As shown in Table 4, the flavonoid glycosides (rutin, isoquercitrin, kaempferol-3-*O*-rutinoside) were transformed into quercetin and kaempferol by extrusion, and especially IEPT. Similarly, previous studies have confirmed that the inhibitory activity of quercetin and kaempferol on α-glucosidase was superior to those of the glycoside derivatives (8, 19). Compared with individual flavonoids, Oboh et al. (44) found that the combination of quercetin and rutin had a synergistic effect on α-glucosidase inhibitory activity.

Not all TBF samples showed higher than 50% inactivation of the α-amylase enzyme within the concentration range studied. Therefore, the IC_50_ value for α-amylase inhibition could not be estimated in this study (Figure 3B). The α-amylase inhibitory activity significantly decreased (*p* < 0.05) under the extrusion processes, except for T160M30 and T70M30. Among the extrusion processes, the most α-amylase inhibitory activity was lost under T100My. It has been determined that individual flavonoids (rutin, isoquercitrin, quercetin) play a major role in the inhibition of α-amylase (45). In addition, the complex produced by the combination of individual flavonoids and α-amylase could cause static quenching of α-amylase *via* non-radiation energy transfer and further inhibit the activity of α-amylase (45). Therefore, we deduced that the inhibitory effect of flavonoid extracts from Tartary buckwheat samples on α-amylase might be the result of the combined effect of multiple active ingredients.

The PTBF obtained with IEPT exhibited strong α-glucosidase inhibitory activity and mild α-amylase inhibitory activity in this study. In view of the fact that strong α-amylase inhibition could cause undesirable effects, such as diarrhea and flatulence (11, 12); PTBF can be used as an ideal α-amylase and α-glucosidase inhibitor and it has great potential in the development of anti-diabetic foods.

### Pearson’s correlations

The Pearson correlation coefficients for the relationship between flavonoid compounds and color parameters, the activities of antioxidant, α-glucosidase, and α-amylase inhibitory of different samples are shown in Table 5. The a* scores significantly correlated with TFC, rutin, isoquercitrin, kaempferol-3-*O*-rutinoside, quercetin, and kaempferol levels (*r* = 0.486, 0.600, 0.453, −0.496, −0.480), whereas the b* scores possessed the opposite tendency. This suggested that the a* and b* scores of different samples are closely related to the individual flavonoid compounds levels. In addition, the antioxidants (DPPH, ABTS, FRAP) significantly correlated with flavonoid levels in Tartary buckwheat samples. Specifically, the antioxidant activity was positively correlated with the levels of quercetin and kaempferol and negatively correlated with TFC, rutin, isoquercitrin, and kaempferol-3-*O*-rutinoside levels. The α-glucosidase inhibitory activity was positively correlated with the levels of quercetin and kaempferol, and negatively correlated with TFC, rutin, isoquercitrin and kaempferol-3-*O*-rutinoside levels, whereas the α-amylase inhibitory activity possessed the opposite tendency. Based on these results, we can conclude that the changes in flavonoid content and composition caused by extrusion have synergistic or antagonistic effects on the color parameters, the activities of antioxidant, α-glucosidase, and α-amylase inhibitory of different samples.

**TABLE 5:** Correlation between flavonoid compounds and color parameters, the activities of antioxidant, α-glucosidase, and α-amylase inhibitory.

	*L* *	*a* *	*b* *	DPPH	ABTS	FRAP	α -glucosidase IC_50_ (μ g/ml)	α -amylase inhibitory activities (%)
Total flavonoids	0.318	0.130	–0.223	0.152	−0.544**	−0.663**	0.508*	0.528**
Rutin	0.305	0.486*	−0.661**	–0.362	−0.877**	−0.972**	0.584**	0.688**
Isoquercitrin	0.248	0.600**	−0.694**	−0.467*	−0.860**	−0.969**	0.493*	0.760**
Kaempferol-3-*O*-rutinoside	0.331	0.453*	−0.670**	–0.328	−0.881**	−0.979*	0.611**	0.660**
Quercetin	–0.386	−0.496*	0824**	0.509*	0.894**	0.944**	−0.581**	−0.681**
Kaempferol	–0.386	−0.480*	0.725**	0.379	0.865**	0.983**	−0.601**	−0.691**

Pearson correlation tests are performed to calculate the correlations between variables. * represents significant difference between data, *p* < 0.05; ** represents extremely significant difference between data, *p* < 0.01.

## Conclusion

This work indicated that the type of extrusion treatment induces a significant effect on the phytochemical composition and color properties of Tartary buckwheat samples and strongly influences its antioxidant, α-glucosidase, and α-amylase inhibitory activities. Extrusion processes have a significant effect on the overall color of Tartary buckwheat samples. The contents of fat, total flavonoids, and flavonoid glycosides decreased upon extrusion, while the aglycone contents and the activities of antioxidant and α-glucosidase inhibition increased significantly. In particular, different aspects of the extrusion treatments (including the temperature and moisture properties) had different effects on the levels of protein and DCI, as well as α-amylase inhibitory activity. Overall, PTBF obtained with IEPT showed particularly high levels of aglycones, strong antioxidant and α-glucosidase inhibition and relatively mild α-amylase inhibitory activity. These findings indicate that PTBF obtained under IEPT could serve as an ideal functional food resource with antioxidant and anti-diabetic potential.

## Data availability statement

The original contributions presented in the study are included in the article/supplementary material, further inquiries can be directed to the corresponding authors.

## Author contributions

ZZ: investigation, data curation, and writing—original draft. XF: methodology and formal analysis. LZu: material and formal analysis. BX: software and formal analysis. MZ: visualization. XY: writing—review and editing. GR: project administration. YY: methodology and writing—review and editing. LZa: supervision and resources. PQ: supervision, conceptualization, and funding acquisition. All authors contributed to the article and approved the submitted version.
